# Differences between Two Methods to Stabilize Supramalleolar Osteotomies in Children—A Retrospective Case Series

**DOI:** 10.3390/children8020086

**Published:** 2021-01-27

**Authors:** Thomas Schlemmer, Reinald Brunner, Bernhard Speth, Johannes Mayr, Erich Rutz

**Affiliations:** 1 Neuroorthopedics, University Children’s Hospital Basel (UKBB), Spitalstrasse 33, 4056 Basel, Switzerland; schlemmerthomas@gmail.com (T.S.); reinald.brunner@ukbb.ch (R.B.); berhard.speth@ukbb.ch (B.S.); johannes.mayr@ukbb.ch (J.M.); 2 Faculty of Medicine, The University of Basel, 4001 Basel, Switzerland; 3 Department of Orthopaedics, The Royal Children’s Hospital Melbourne, Murdoch Children’s Research Institute (MCRI), Melbourne, VIC 3052, Australia

**Keywords:** supramalleolar osteotomy, fixation methods, locking compression plate, LOS, Kirschner wire, radiographic consolidation

## Abstract

Supramalleolar osteotomy (SMO) in pediatric patients can be fixed in various ways. We analyzed the records of 77 pediatric patients (124 SMOs) aged ≤16 years. In 56 patients (96 SMOs), K-wires were used to stabilize SMOs (WF group), while 21 patients (28 SMOs) were treated with locking compression plates (LCPs; PF group). We recorded time to radiographic consolidation, rate of complications, length of hospital stay (LOS), and time to implant removal. Mean time to radiographic consolidation of SMOs was 7.2 weeks in the WF group and 11.1 weeks in the PF group. Complication rate in the WF group was 10.7%. LOS was similar in the two groups (7.0 days in the WF group vs. 7.3 days in the PF group). K-wire stabilization resulted in a shortened interval until consolidation of osteotomies, but children were required to use a cast. Stabilization of SMOs with LCPs facilitated early mobilization and functional rehabilitation with no need to apply a cast. In conclusion, both methods provided safe fixation of SMOs with a low rate of complications. K-wire stabilization combined with a cast achieves fast consolidation of SMOs. We recommend SMO stabilization with angular stable LCPs in patients with muscular weakness or spasticity in whom early mobilization and physiotherapy are necessary to prevent loss of muscle power, muscle function, and bone mass.

## 1. Introduction

Children suffering from cerebral palsy frequently show pathologic internal or external torsion of the tibia [1]. Children suffering from cerebral palsy (CP) and internal or external torsion of the tibia frequently face impaired mobility, joint dysfunction, and impairment of activities of daily living resulting in problems not only for the child but also for families, caregivers, and nursing staff. Impaired mobility has massive long-term implications for the child in terms of social integration, pain, and physical as well as emotional well-being. Surgical correction of such torsions improves frontal plane knee moments and helps to restore ankle power in inward torsion of the tibia [2,3,4,5]. Moreover, correction of pathologic internal tibial torsion improves kinematic and kinetic gait alterations associated with intoeing gait [3,6] and improves functional parameters of gait analysis in patients with cerebral palsy [7].

Rotational deformities of the tibia can cause severe impairment of ambulation, dysfunction of joints, and arthritic changes of joints [8,9,10]. Limb spasticity in children suffering from CP can be ameliorated by sequential botulinum toxin injections into affected muscles and bracing [11]. Er et al. and Inan et al. reported promising results for surgical correction of external and internal rotational deformities of the tibia in children suffering from CP [8,9,10].

Supramalleolar osteotomy (SMO) may involve various devices such as drills, Gigli saw, or oscillating saws [10,12]. Even percutaneous drill holes and closed osteoclasis have been applied to create percutaneous SMOs [10]. Techniques to stabilize SMOs may involve Kirschner wires (K-wires), external fixators, intramedullary nails, locking plates, or screws [12,13,14,15,16]. In children, correctional tibial osteotomy is preferentially done at the distal (supramalleolar) metaphysis since proximal tibia osteotomy is associated with a higher rate of major complications [17].

**Figure 1:**
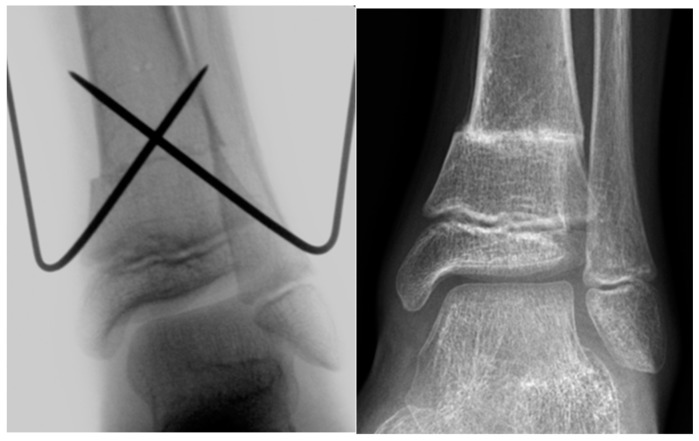
Intraoperative X-ray image and X-ray image obtained at 2 months after SMO fixed with K‑wires in an 11-year-old male patient with increased external torsion of the tibia.

**Figure 2:**
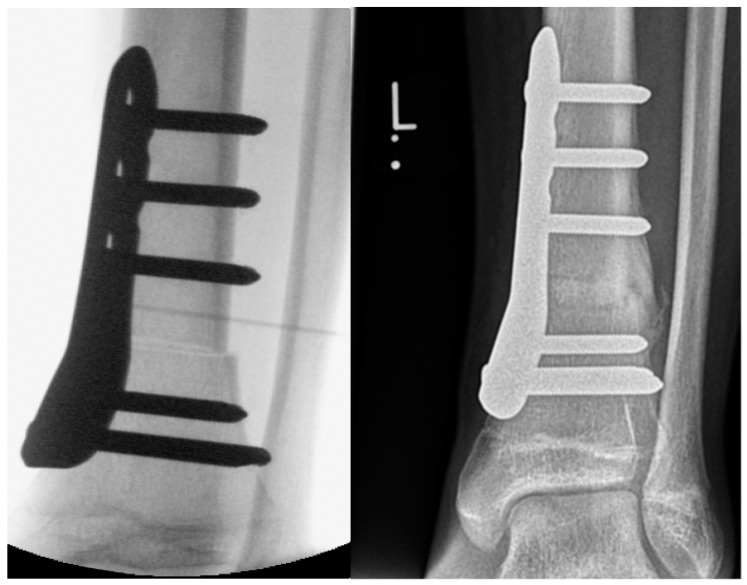
Intraoperative X-ray image and X-ray image obtained 3 months after SMO fixed with an angular stable plate in a 14-year-old male patient with increased external torsion of the tibia.

De Roode et al. reported higher rates of complications in patients who underwent plate fixation of SMO [13]. The authors applied crossed K‑wire stabilization followed by postoperative cast immobilization of the lower leg as well as SMO fixation with nonangular stable plates, followed by lower-leg cast immobilization. At our institution, we use both K-wires (Figure 1) and locking compression plates (LCP; Figure 2) to stabilize SMOs in children.

Indications for plate fixation include impaired bone quality due to concomitant diseases or dependence on early weight bearing due to limited motor function, e.g., in cerebral palsy, as well as inability of partial weight bearing. Plate fixation is also indicated in patients undergoing other surgical interventions, as well as in patients requiring early functional aftercare or those who are no candidates for cast application, as in simultaneous lengthening of the tibia. In otherwise healthy patients, SMO stabilization with K-wires and plaster cast is used.

In SMOs stabilized with a plate, we use an angular stable LCP, which is in contrast to the nonangular plate used by de Roode et al. [13]. Angular stability of the LCP provides higher primary stability of the osteotomy compared to K-wire fixation or nonlocking plates. The Pediatric LCP Condylar 90-Degree Plate (Johnson & Johnson, Synthes, Oberdorf, Switzerland) can be used to stabilize SMOs as well as supracondylar distal femur osteotomies [18,19] due to the anatomic design of the plate. This implant allows weight bearing up to 35 kg if 3.5 mm screws are used and up to 70 kg if 5.0 mm screws are used. LCP dimensions chosen depend on the patient’s age, height, and body weight.

We use plate fixation of SMOs in patients suffering from more complex disorders. The higher rate of complications associated with plate stabilization versus K-wire fixation reported by de Roode et al. [13] prompted us to scrutinize our strategy. Thus, we retrospectively evaluated SMO fixation either with K-wires followed by cast application or with LCP with respect to radiographic consolidation of the osteotomy, number and severity of complications, and LOS. We also assessed duration of cast immobilization in children treated with K-wire stabilization of SMO and assessed time to full weight bearing in both groups. Additionally, we aimed to examine the type of accompanying diseases, indications for osteotomy, type of additional surgical procedures applied, and complication rates of both methods of SMO stabilization. We also aimed to evaluate the degree of rotational correction observed at follow-up and to establish recommendations for the efficient and safe treatment of rotational deformities of the tibia in children suffering from neurological disorders.

## 2. Patients and Methods

### 2.1. Patients

We included all patients who underwent SMOs due to any tibial malalignment (varus, valgus, and internal or external torsion) and were no older than 16 years at the time of surgery. For inclusion in the study, complete patient records and radiographic follow-up data at least until SMO consolidation in the group undergoing K-wire fixation and until plate removal in the group undergoing plate fixation were required. We included children with intoeing and outtoeing gait problems with tibial internal or external torsion treated at our institution between January 2010 and December 2016.

Patients who underwent SMO fixation with nonangular stable plates were excluded from this study. We also excluded children with intoeing and outtoeing gait whose gait dysfunction was related to foot pathology or femoral torsional malalignment. We further excluded children who had undergone previous tibial osteotomy or had sustained acute fracture of the tibia, as well as children suffering from tibial bone cyst or tumor.

Overall, 77 patients (124 SMOs) met the inclusion criteria. Of these, 56 patients (96 SMOs), underwent K-wires stabilization of SMO (WF group), while 21 patients (28 SMOs) were treated with LCPs (PF group). Mean age of patients at the time of surgery in the WF group was 9.9 years (range: 5.2–14.7 years; SD: 1.7 years). In the PF group, the mean age of children was 12.2 years (range: 2.8–16 years; SD: 3.1 years). We treated 45 (80.4%) boys and 11 (19.6%) girls with K-wire fixation and 7 (33.3%) girls and 14 (66.7%) boys with LCP fixation.

Table 1 shows the additional surgical procedures, main diagnoses, and concomitant diseases in patients of the WF group.

In the PF group, indications for surgery were more varied and complex (Table 2).

### 2.2. Ethical Approval

After receiving approval of the study protocol by the Ethics Committee of Northwestern and Central Switzerland (EKNZ 304/08 amendment 2018-00168), we reviewed the records of 185 SMOs performed between January 2010 and December 2016 at our institution. The study was carried out in accordance with the World Medical Association Declaration of Helsinki.

### 2.3. Surgical Procedures

We chose the indications and surgical technique for SMO in line with the recommendations published by Mulhern et al. [20].

**Figure 3:**
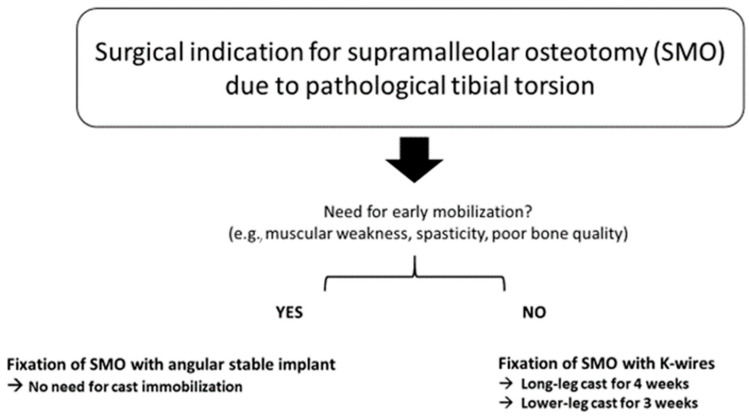
Management algorithm for pathological tibial torsion in children.

The treatment algorithm we used in our patients is shown in Figure 3.

In the WF group, SMOs were performed using a drill and chisel in 90 of 96 (93.8%) SMOs or an oscillating saw in 6 of 96 (6.3%) SMOs.

In the PF group, all SMOs involved an oscillating saw. In 56 patients (96 SMOs), we used K-wires and in 21 patients (28 SMOs), we applied LCPs. In 16 SMOs, we used the Pediatric LCP Condylar 90-Degree Plate (Johnson & Johnson, Synthes, Oberdorf, Switzerland). In the remaining patients, other angular stable implants were used according to the surgeon’s choice. While patients in the WF group received a cast after the operation, no cast was required in patients in the PF group unless this was required because of other surgeries performed simultaneously. In the WF group, 40 patients underwent bilateral SMOs, while 16 patients had unilateral SMOs. In the PF group, 7 patients underwent bilateral and 14 unilateral SMOs.

### 2.4. Study Variables

We analyzed time to radiographic consolidation (defined as bridging callus of at least three of the four cortices in the anteroposterior and mediolateral plain X-ray images in accordance with the definition of Inan et al. [10]), rate and type of postoperative surgical complications, and LOS after surgery. We rated postoperative complications according to Clavien–Dindo classification of surgical complications [21]. Additionally, we determined time to full weight bearing, duration of long-leg or lower-leg cast therapy in the WF group, time to implant removal, as well as the degree of torsional correction.

To limit the influence of confounding variables and reduce bias, we described the diseases and concomitant disorders in the two study groups separately and opted against comparing the groups due to different patient characteristics in terms of age, type of comorbidities, types of additional surgical procedures applied, and variable follow-up intervals. Data were analyzed descriptively and presented in tables.

### 2.5. Statistical Analyses

We used independent-samples *t*-test to assess the difference of torsional correction between the group of patients who suffered a complication and the group who did not. Data were normally distributed in both groups. A *p*-value < 0.05 was considered significant.

## 3. Results

### 3.1. Radiographic Consolidation

In the WF group, SMO consolidation was achieved after a mean of 7.2 weeks (range: 4–15 weeks; SD: 1.9 weeks). In the PF group, radiographic consolidation of osteotomy was achieved after a mean of 11.1 weeks (range: 6–28 weeks; SD: 5.5 weeks).

In the WF group, we were unable to analyze the influence of using an oscillating saw in comparison to drilling for the osteotomy because only 6 of 96 (6.3%) SMOs were performed in this way. In contrast, all SMOs in the PF group involved the use of an oscillating saw.

### 3.2. Rate and Type of Complications

Overall complication rates were 26.0% in the WF group and 10.7% in the PF group. In the WF group, displacement of a K-wire was the most frequent complication, occurring in 13 of the 96 (13.5%) SMOs. However, displaced K-wires did not require further surgical intervention or prolonged cast stabilization. In 9 of 96 (9.4%) SMOs in the WF group, superficial wound infection occurred. Oral antibiotic treatment for 10 days was required in 2 patients, but no surgical intervention was necessary. All complications were grade I complications according to the Clavien–Dindo classification of surgical complications [21]. One patient experienced a compartment syndrome in the lower leg after K-wire stabilization of the SMO. This major complication (grade III complication according to Clavien–Dindo classification [21]) required fasciotomy and staged wound closure. One patient in the WF group sustained a fracture at the site of the SMO due to an injury 8 weeks after surgery. The fracture was treated conservatively by a lower-leg cast and healed uneventfully. In 2 of 96 (2.1%) SMOs, internal torsion persisted.

In the PF group, osteolysis at the medial cortex of the fibula occurred in 1 of 28 (3.6%) SMOs due to an excessively long screw. Another patient in the PF group experienced a fissure at the SMO site after plate removal, which healed without any treatment. The fissure was detected coincidentally due to callus formation evidenced in a postoperative plain X-ray image, and the patient was asymptomatic at all times. In 1 of 28 (3.6%) SMOs in the PF group, superficial wound infection (classified as grade I complication according to the Clavien–Dindo classification of surgical complications [21]) occurred. The infection was successfully treated with oral antibiotics given for 10 days.

The degree of torsional correction in all patients who suffered a complication was 18.8°, while the degree of correction in the patients without a complication amounted to 18.7° (*p* = 0.467). Thus, we observed no correlation between the degree of correction and occurrence of a complication.

### 3.3. Length of Hospital Stay

LOS after surgery is shown in Table 3.

### 3.4. Duration of Cast Immobilization

All SMOs stabilized with K-wires were protected with plaster casts for a mean duration of 7.1 weeks (range: 6–16 weeks; SD: 1.6 weeks). A long-leg cast was applied for a mean of 4.1 weeks (range: 4–12 weeks), followed by a lower-leg cast for a mean period of 3.0 weeks (range: 2–5 weeks; SD: 1.6 weeks). The lower leg of one patient was immobilized in a vacuum boot for an additional 12 weeks after removal of the lower-leg cast due to incomplete SMO consolidation.

In the PF group, 5 SMOs required a lower-leg cast without weight bearing for 6 weeks due to other surgeries performed simultaneously.

### 3.5. Time to Implant Removal.

K-wires and plates were removed after mean periods of 4.4 weeks (range: 3–7 weeks; SD: 0.9 weeks) and 50.3 weeks (range: 19–112 weeks; SD: 20.4 weeks), respectively.

### 3.6. Degree of Correction

Mean torsional correction in the WF group was 18.7° (range: 10°–30°; SD: 3.9°).

In the PF group, we achieved a mean torsional correction of 21.2° (range: 10°–45°; SD: 8.9°).

Additional osteotomy of the fibula was avoided in all patients except one patient in PF group.

### 3.7. Follow-Up Period

Mean intervals of clinical and radiographic follow-up in the WF group amounted to 9.1 months (range: 2–30 months; SD: 6.9 months). In PF group, the mean follow-up interval was 30.1 months (range: 9–69 months; SD: 18.2 months).

## 4. Discussion

Published studies comparing the two techniques of SMO fixation are very rare. In 2013, De Roode et al. reported that stabilizing SMOs with nonangular stable plates is less reliable than using K-wires and is associated with more complications [13]. In contrast to Roode et al., we used angular stable plates for fixation of SMO and observed a lower rate of complications when compared to their findings obtained with nonangular stable implants [13]. This is even more remarkable since the patients in the PF group had more severe deformities and disorders and thus required surgeries in part (Table 2).

Angular LCP fixation offers the advantage of early weight bearing without the need to apply a postoperative cast. A drawback of this approach is the larger scar resulting from plate fixation and a second surgery for plate removal.

### 4.1. Time to Radiographic Consolidation

Our retrospective study proved that the mean time to radiographic consolidation in the WF group was as short as 7.2 weeks. Savva et al. reported a mean time to union of 12 weeks after unilateral SMO stabilized with staples [22]. Nevertheless, the authors did not specify their definition of “union,” and this was no primary endpoint in their study. We did not find any literature concerning consolidation of SMOs after K-wire fixation. A possible explanation for the fast radiographic healing after SMO fixation with K‑wires is the relative instability of the wires, which promotes callus formation [23].

### 4.2. Complication Rates

Overall complication rates were low in both the WF group and PF group. Most complications were minor. A single patient in the WF group experienced compartment syndrome of the lower leg as a major complication. One patient in the PF group experienced a fissure at the site of the healed osteotomy after plate removal. In a series of 91 SMOs stabilized with AO-ASIF T plates (Johnson & Johnson, Synthes, Oberdorf, Switzerland), Selber et al. reported three late complications, i.e., fracture at the site of osteotomy after plate removal, nonunion, and growth arrest of the distal tibial physis [14]. This compares well to our findings in the PF group.

The incidence of infections in the WF group was similar to that reported in a study investigating pin-tract infections in children [24]. Different definitions of pin-tract infection have been proposed; we defined it as redness of the entry point of the pin. Fluid secretion or antibiotic treatment was no prerequisite to confirm an infection. The same definition was used for wound infections in the PF group. Surgical revision to treat wound infection was not required in any case, and all wound infections healed without sequelae. The most severe complication in the PF group was osteolysis of the medial cortex of the fibula in one patient. Osteolysis was asymptomatic, and the fibula remodeled after implant removal.

In all patients who experienced postoperative K-wire displacement, the K‑wires had penetrated both cortices as visualized in intraoperative X-ray images. In three SMOs, a combination of K-wire displacement and superficial infection at the entry point occurred. Approximately 30% of wound infections were accompanied by K-wire displacement, and approximately 25% of all displaced K-wires were accompanied by superficial infection. The statistical power of our investigation was too low to identify any clear risk factors for K-wire displacement.

### 4.3. Length of Hospital Stay

Patients with bilateral SMOs fixed with K-wires had a somewhat shorter LOS than those in whom LCPs were used. However, it should be borne in mind that the criteria for hospital discharge in these patient groups differed. Patients with K-wire fixation had to be able to sit in a wheelchair, whereas patients with plate fixation had to be able to walk with crutches before leaving the hospital.

Both primary and concomitant diseases of patients in the PF group were highly heterogeneous. Thus, we analyzed these patients per subgroup, which precluded statistical evaluation due to the small subgroup populations.

### 4.4. Time to Mobilization

Bilateral SMOs fixed with K-wires initially required long-leg casts, which limited mobilization. Especially in patients undergoing bilateral correction, the use of the LCP facilitated early mobilization. The longer time to consolidation in the PF group was negligible in our view as it had no long-term disadvantage for the patient. Full weight bearing due to the stability of the plate was possible even if the osteotomy had not healed completely due to the high angular stability of the plate. We do not advocate sequential correction of bilateral deformities because the rehabilitation period would be twice as long.

We avoided osteotomy of the fibula in all our SMO patients. However, Andrisevic et al. observed transient subluxation of the proximal tibiofibular joint after performing isolated SMO of the tibia and reported that remodeling of the fibula corrected tibiofibular subluxation in all patients [1].

### 4.5. Surgical Time and Expenses

Since we focused primarily on the outcome of the SMOs, we did not address the duration of surgical intervention in this study. In many patients in the PF group, duration of SMO fixation could not be assessed because other surgeries were conducted at the same time. De Roode et al. [13] reported surgery time to be longer in SMO patients undergoing plate fixation than in those undergoing K-wire fixation. However, patients undergoing K-wire fixation received a long-leg cast during the ongoing general anesthesia, thus prolonging the total anesthetic procedure.

In terms of overall expenses, implant costs for LCPs greatly exceed those for K-wires.

### 4.6. Time to Implant Removal

The decision to remove the LCP was determined by radiographically confirmed consolidation of the osteotomy. There are no strict rules whether to remove the plate after consolidation, except in cases where the plate causes discomfort to the patient. In contrast, after K-wire fixation all metal parts have to be removed as early as reasonably possible. In our study, optional plate removal allowed us to combine the intervention with other interventions requiring anesthesia, as, e.g., soft tissue procedures in patients with cerebral palsy.

### 4.7. Follow-Up Time after Plate or K-Wire Stabilization of SMOs

The large difference in the follow-up time between the two groups was caused by the nature of the underlying disease. While patients in the WF group needed no further medical checkups upon completion of SMO, many patients of the PF group suffered from neuromuscular disorders and were thus seen for follow-up investigations on a regular basis.

### 4.8. Recommendations

In addition to performing safe and effective correction of the deformity, we consider it an important goal in the aftercare of these patients to minimize time of immobilization and maximize the patients’ comfort during the healing period. Based on our data, we recommend the use of K-wire fixation for unilateral correction in otherwise healthy patients below the age of 10 years. These patients tend to be able to walk with crutches with weight bearing on the nonoperated leg and thus do not depend on wheelchairs for 4 weeks. A second surgery for implant removal is not necessary. In older patients, the use of angular stable plates is reasonable due to the limited stability of K-wires in adolescent patients. In patients with concomitant diseases, especially those with neuromuscular problems such as cerebral palsy or similar syndromes with muscular weakness and/or spasticity, we recommend the use of an angular stable plate in order to provide primary stability and early mobilization of patients to prevent loss of muscle function and power.

In our study, we avoided to apply a cast after plate fixation of SMOs and obtained healing of the osteotomies in all patients. Kolp et al. applied a lower-leg cast for 4–5 weeks after SMO fixation with four-hole 3.5 mm 90° LCP plates (Johnson & Johnson, Synthes, Oberdorf, Switzerland), which is in contrast to our treatment regimen [16].

Since there is no significant disadvantage besides the necessary implant removal in patients with plate fixation and early mobilization is possible, use of angular stable plates in otherwise healthy patients with bilateral tibia torsion malalignment as well as in patients with concomitant diseases can be a treatment option. Moreover, in patients who prefer early weight bearing, unilateral fixation with an angular stable plate is a valid approach.

### 4.9. Limitations of Study

Our findings must be interpreted with caution due to the retrospective nature and the small number of patients in the PF group. In addition, the follow-up intervals in the two groups differed. Due to the variable patient characteristics and the higher rate of accompanying diseases and potentially poorer bone quality in patients of the PF group, we decided not to compare findings between groups to exclude selection bias. Because we did not analyze the preoperative and postoperative gait in all patients, we were unable to provide results of the functional gait analysis.

Er et al. reported that in the long term, tibial external rotation might increase postoperatively [8,9]. Due to the lack of long-term results, we cannot exclude possible long-term changes of tibial rotational outcomes.

## 5. Conclusions

Fixation of SMO with K-wires and application of a cast resulted in radiographic consolidation of osteotomies within 7 weeks. Our study confirmed that SMO fixation with an angular stable LCP permits early mobilization without the need of a cast. This approach resulted in a low complication rate. Since no casts are required after SMO stabilization with an angular stable locking plate, patients are able to ambulate with full weight bearing. A drawback is the surgery needed for plate removal. Based on our findings, we recommend to use K-wire stabilization for unilateral SMOs performed in otherwise healthy children below the age of 10 years. In older children and children suffering from bilateral tibial torsion or more complex accompanying disorders, we consider LCP stabilization of SMOs a promising treatment option.

## Figures and Tables

**Table 1:** Additional surgical procedures and concomitant diseases in the underwent K-wires stabilization of supramalleolar osteotomy (WF group) (*n* = 56 patients; 96 supramalleolar osteotomies (SMOs)).

Additional Surgical Procedures	Number of Patients
Distal medial epiphysiodesis femur due to genu valgum	1
Shortening of Achilles tendon	1
Proximal medial epiphysiodesis tibia due to genu valgum	1
Total	3
Main Diagnosis	Number of SMOs
External tibial torsion	56
Internal tibial torsion	40
Total	96
Concomitant Diseases	Number of Patients
Genu valgum	3
Congenital club foot	2
Post-traumatic deformity	2
Pes planovalgus	2
Cerebral palsy	2
Genu varum	3
Total	14

**Table 2:** Additional surgical procedures and concomitant diseases in the treated with locking compression plates (PF group) (*n* = 21 patients; 28 SMOs).

Additional Surgical Procedures (*n* Represents Procedure)	Numbers
Resection of multiple osteochondromas	3
Lengthening of tibia	1
Calcaneus lengthening osteotomy	1
Talonavicular arthrodesis	2
Distal femur osteotomy	3
Subtalar arthrodesis	1
Implant removal	7
Calf lengthening	2
Arthroscopy knee joint	1
Fibular osteotomy	1
Mid foot osteotomy	1
Epiphysiodesis proximal tibia	1
Shortening of M. tibialis anterior tendon	1
Lengthening of M. tibialis posterior tendon	1
Total number of additional procedures	26
Total number of patients with additional surgical procedures	12
Main diagnosis	Numbers
External tibial torsion	14
Internal tibial torsion	14
Total	28
Concomitant diseases	Numbers
Multiple osteochondroma	3
Cerebral palsy	3
Pes planovalgus	2
Spina bifida	4
Club foot	2
Leg length discrepancy (post-traumatic)	1
Arthrogryposis multiplex congenita	1
Hereditary spastic paraplegia	2
Total	18

**Table 3:** Length of hospital stay (LOS) of the various subgroups.

Subgroup	Mean LOS (Days)
All patients with K-wire fixation (*n* = 56)	7.0 (range: 3–33; SD: 3.2)
All patients with plate fixation (*n* = 21)	7.3 (range: 3–14; SD: 3.3)
Otherwise healthy SMO patients treated with K-wire fixation	7.1 (range: 5–33; SD: 3.3)
Otherwise healthy SMO patients treated with plate fixation	6.8 (range: 5–12; SD: 2.2)
SMO patients treated with bilateral K-wire fixation	6.8 (range: 4–14; SD: 1.8)
SMO patients treated with bilateral plate fixation	7.4 (range: 4–14; SD: 3.4)
SMO patients treated with unilateral K-wire fixation	8.3 (range: 5–33; SD: 6.7)
SMO patients treated with unilateral plate fixation	7.2 (range: 3–15; SD: 3.3)

LOS = length of hospital stay; SD = standard deviation; SMO = supramalleolar osteotomy.

## Data Availability

The data presented in this study are available from the corresponding author on reasonable request.
